# Zirconium-89-Oxine Cell Tracking by PET Reveals Preferential Monocyte Recruitment to Cancer and Inflammation over Macrophages

**DOI:** 10.3390/ph18060897

**Published:** 2025-06-15

**Authors:** Sho Koyasu, Hannah A. Minor, Kingsley O. Asiedu, Peter L. Choyke, Noriko Sato

**Affiliations:** 1 Molecular Imaging Branch, National Cancer Institute, National Institutes of Health, Bethesda, MD 20892, USA; sho@kuhp.kyoto-u.ac.jp (S.K.); hminor2@student.umgc.edu (H.A.M.); kingsley.asiedu@duke.edu (K.O.A.); pchoyke@mail.nih.gov (P.L.C.); 2 Department of Diagnostic Imaging and Nuclear Medicine, Graduate School of Medicine, Kyoto University, Kyoto 606-8507, Japan; 3 School of Arts and Sciences, University of Maryland Global Campus, Adelphi, MD 20783, USA

**Keywords:** monocyte, macrophage, cell tracking, ^89^Zr-oxine, positron emission tomography, cell-based therapy, cell trafficking, inflammation, cancer

## Abstract

**Background/Objectives:** Cell-based therapies have become increasingly important in the treatment of cancers and inflammatory diseases; however, therapies utilizing monocyte–macrophage lineage cells remain relatively underexplored. Non-invasive cell tracking allows a better understanding of the fate of such cells, which is essential for leveraging their therapeutic potential. Here, we employed a Zirconium-89 (^89^Zr)-oxine cell labeling method to compare the trafficking of monocytes and macrophages in vivo. **Methods:** Mouse bone marrow-derived monocytes and macrophages were each labeled with ^89^Zr-oxine and evaluated for their viability, radioactivity retention, chemotaxis, and phagocytic function in vitro. Labeled cells were intravenously administered to healthy mice and to murine models of granuloma and syngeneic tumors. Cell migration was monitored using microPET/CT, while cell recruitment to the lesions was further assessed via ex vivo biodistribution and flow cytometry. **Results:** Labeled cells exhibited similar survival and proliferation to unlabeled cells for up to 7 days in culture. While both maintained phagocytic function, monocytes showed higher CCL2-driven chemotaxis compared to macrophages. ^89^Zr-oxine PET revealed initial cell accumulation in the lungs, followed by their homing to the liver and spleen within 2–24 h, persisting through the 5-day observation period. Notably, monocytes trafficked to the liver and spleen more rapidly than macrophages. In both inflammation and cancer models, monocytes demonstrated higher accumulation at the lesion sites compared to macrophages. **Conclusions:** This study demonstrates the usefulness of ^89^Zr-oxine PET in tracking monocyte–macrophage lineage cells, highlighting their distinct migration patterns and providing insights that could advance monocyte-centered cell therapies.

## 1. Introduction

Monocyte–macrophage lineage cells are key components of the mononuclear phagocyte system and serve as critical effectors and regulators of inflammation, the innate immune response, and the clearance of apoptotic cells [1,2]. Monocytes originate in the bone marrow from common myeloid progenitor cells, and can differentiate into macrophages or inflammatory dendritic cells (DCs) during inflammation, as well as under steady-state conditions [1,2,3]. Macrophages are tissue-resident phagocytic cells comprised of a mix of embryonically derived and bone marrow-derived cells, with different tissues having varying compositions and turnover rates of selected macrophage subpopulations. Subsets of tissue-resident macrophages include microglia, dermal macrophages, and splenic marginal zone and metallophilic macrophages [1,2,3]. These primarily derive from embryonic progenitors in the yolk sac and fetal liver during embryonic development, while bone marrow hematopoietic stem cells also contribute to the macrophage pool [1,4]. Macrophages exhibit unique plasticity and can polarize into two primary distinct phenotypes: M1 and M2. Interferon and Toll-like receptor signaling can induce differentiation into the anti-tumorigenic, pro-inflammatory M1 phenotype, and interleukin (IL)-4 and IL-13 signaling can induce the M2 phenotype, which promotes anti-inflammatory, pro-tumorigenic microenvironment and angiogenesis [2,5,6,7,8,9]. 

Cell-based therapies, typically employing stem cells or lymphocytes, are gaining importance in the treatment of cancer and inflammatory diseases [10,11]. However, applications involving monocyte–macrophage lineage cells have only recently emerged [2,5,12,13,14]. For instance, a macrophage-based cell therapy has been reported to improve experimental chronic liver injury, whereas treatments using whole bone marrow or purified macrophage precursors in the marrow fail to do so [15]. Another potential application of monocyte–macrophage lineage cells is to employ them as live carriers of therapeutic nanoparticles, enabling drug delivery to otherwise inaccessible regions, such as hypoxic tumor regions [16,17]. Monocyte-based therapy has also shown therapeutic potential [13,14]. For instance, in a rat stroke model, systemic infusion of CD14⁺ human monocytes significantly reduced infarct size and improved functional recovery, contributing to neuronal protection and brain repair [14]. More recent studies indicate that CD14⁺ monocytes mediate the expression of effector proteins that prevent neuronal death following glucose and oxygen deprivation. Interestingly, CD14⁺ monocytes from human umbilical cord blood appear more effective than those derived from adult peripheral blood in these studies [18,19].

However, it is known that the majority of intravenously administered monocyte–macrophage lineage cells are sequestered by the reticuloendothelial system (lung, liver, and spleen) before reaching target sites, resulting in low homing efficiency [20]. Traditionally, cell distribution has been assessed by tissue biopsy or, in preclinical settings, by autopsy and tissue extraction, which are time-consuming and often require large numbers of animals. These methods are invasive and/or provide only endpoint data; hence, there is a strong need for non-invasive visualization of the infused cells to their fate. Such insights are critical for improving the efficacy of monocyte–macrophage-based therapies.

The Zirconium-89 (^89^Zr)-oxine complex cell labeling method has been developed for in vivo cell tracking by positron emission tomography (PET) imaging [21,22]. ^89^Zr has a relatively long half-life of 3.27 days, making it suitable for tracking cells in vivo up to two weeks. Furthermore, compared to other radionuclide imaging methods such as single-photon emission computed tomography, PET imaging offers at least 10-fold higher sensitivity [23], enabling lower radiation exposure to labeled cells. We and others have successfully examined trafficking of diverse cell types in mice and non-human primates [21,24,25,26,27,28,29,30], as well as in humans [31]. In the present study, we employed ^89^Zr-oxine labeling and PET imaging to compare the distribution and homing efficacy of monocytes and macrophages under normal, inflammatory, and cancerous conditions.

## 2. Results

### 2.1. Bone Marrow-Derived Monocytes and Macrophages Exhibit Distinct Surface Marker Profiles

We first analyzed the surface marker expression of monocytes and macrophages generated from the bone marrow. Both cell types expressed high levels of CD11b and were negative for Ly6G, a hallmark of the monocyte–macrophage lineage cells (Figure S1). Generated monocytes consisted of Ly6C^high^ and Ly6C^intermediate^ subsets, contained CD11c^high^ cells, and expressed high levels of CD62L but low levels of the macrophage marker F4/80. In contrast, bone marrow-derived macrophages showed significantly higher levels of F4/80 and CD80, consistent with mature macrophages. This was further supported by the lack of CD62L expression. Additionally, macrophages exhibited markedly lower levels of C-C chemokine receptor type 2 (CCR2) compared to monocytes.

### 2.2. Labeling with ^89^Zr-Oxine Complex Does Not Interfere with the Viability or Proliferation of Monocytes and Macrophages

**Figure 1:**
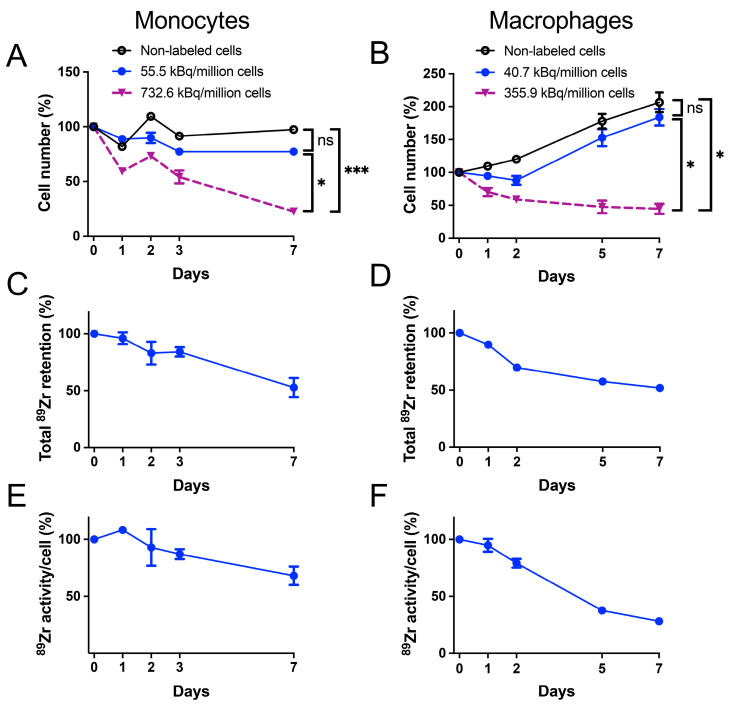
Viability and ^89^Zr retention of ^89^Zr-oxine-labeled monocytes and macrophages. (**A**,**B**) Monocytes (**A**) and macrophages (**B**) were labeled with ^89^Zr-oxine at indicated doses and monitored for viability over 7 days. Cells labeled at lower doses (55.5 kBq/million monocytes and 40.7 kBq/million macrophages) maintained viability and cell number changes comparable to non-labeled controls. In contrast, high-dose labeling significantly impaired cell survival (*n* = 2, *: *p* < 0.05, ***: *p* < 0.001, repeated-measures two-way ANOVA). (**C**,**D**) The decay-corrected total ^89^Zr activity remained over 50% of the initial values in both monocytes (**C**) and macrophages labeled at lower doses through day 7. (**E**,**F**) Specific activity of monocytes (**E**) was maintained at over 68.1% of the initial value, whereas that of macrophages (**F**) declined to 28.2%, reflecting their greater proliferation than monocytes. All data are presented as mean ± standard deviation.

To ensure that radiolabeling does not alter the function of the cells, we labeled monocytes and macrophages with a ^89^Zr-oxine complex and examined the effects on viability, proliferation, and radioactivity retention. Based on our previous studies, we anticipated that labeling doses around approximately 37 kBq/million cells were unlikely to cause cellular radiotoxicity and were suitable for PET imaging [21,24,32]. The cells were also labeled at higher doses to further evaluate radiotoxicity. Monocytes and macrophages labeled at 55.5 kBq/million cells and 40.7 kBq/million cells, respectively, demonstrated preserved viability and proliferation up to 7 days post-labeling. In contrast, viability decreased significantly when higher doses of 732.6 kBq/million monocytes or 355.9 kBq/million macrophages were used (Figure 1A,B). Radioactivity retention remained above 50% through day 7 in both cell types (52.7% for monocytes and 51.8% for macrophages on day 7) (Figure 1C,D). The activity per cell (i.e., specific activity) decreased more in macrophages (28.1% on day 7), likely due to their higher proliferation and dilution of labeled ^89^Zr than monocytes (68.1% on day 7) (Figure 1E,F). Based on these findings, we used a ^89^Zr-oxine labeling dose of approximately 37 kBq/million cells for all of the following experiments.

### 2.3. ^89^Zr-Oxine Labeling Does Not Interfere with Chemotaxis and Phagocytic Functions of Monocytes and Macrophages

**Figure 2:**
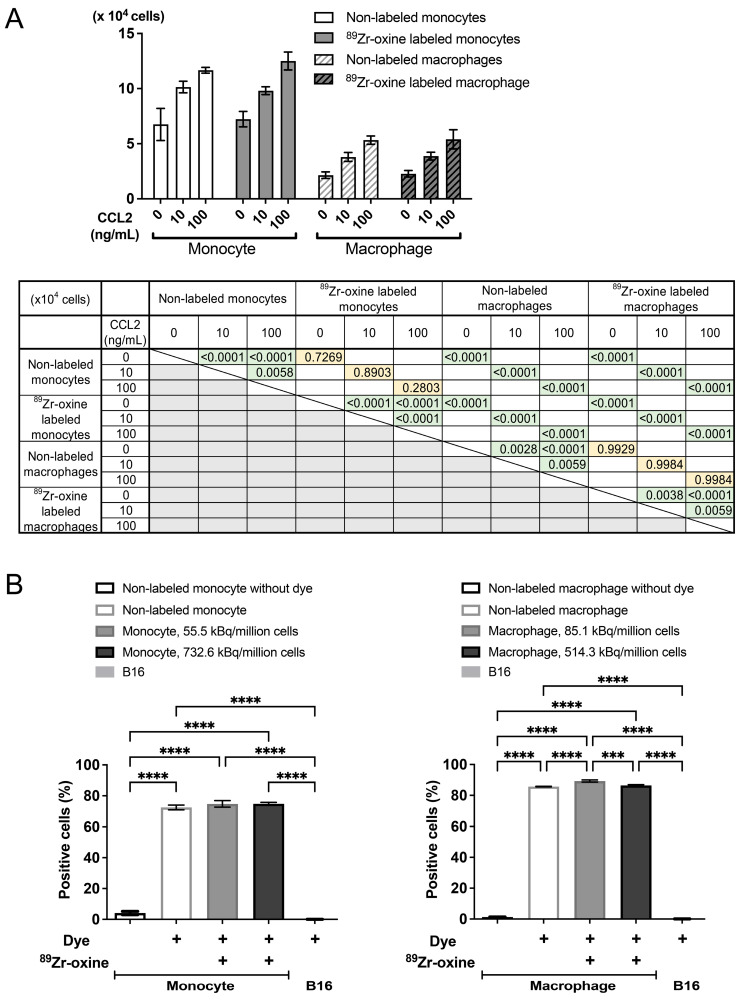
^89^Zr-oxine-labeled monocytes and macrophages maintain their ability to migrate and phagocytize. (**A**) A half million non-labeled or ^89^Zr-oxine-labeled monocytes (55.5 kBq/million cells) or macrophages (38.1 kBq/million cells) were seeded in the upper chambers of transwells and media containing the indicated doses of CCL2 were placed in the lower chambers. After 24 h of culture, the cells that had migrated to the lower chambers were counted. The labeled and non-labeled monocytes and macrophages migrated toward CCL2 in a CCL2-dose dependent manner (*n* = 4). Monocytes demonstrated greater chemotaxis than macrophages. Statistical significance from a two-way ANOVA is summarized in the accompanying table. (**B**) Phagocytosis was assessed using fluorescent dye-conjugated zymosan particles. ^89^Zr-oxine labeling at the indicated doses did not impair the phagocytic capacity of either monocytes (left) or macrophages (right), even at high labeling doses. B16 melanoma cells served as a non-phagocytotic cell control. The percentages of cells phagocytosing the fluorescent dye-conjugated particles were determined by flow cytometry (*n* = 3. ***: *p* < 0.001, ****: *p* < 0.0001 by a two-way ANOVA). All data are presented as mean ± standard deviation.

To determine if ^89^Zr-oxine-labeled cells retain their functional properties, labeled monocytes and macrophages were evaluated for their chemotaxis and phagocytosis functions. A transwell system was utilized to assess chemotaxis. Both labeled and unlabeled monocytes and macrophages migrated through the transwell filter toward chemokine (C-C motif) ligand 2 (CCL2) in a CCL2 dose-dependent manner (Figure 2A). Monocytes had significantly higher chemotactic activity than macrophages, irrespective of ^89^Zr-oxine labeling status. For assessing phagocytic function, the cells were cultured with bioparticles conjugated with zymosan, a fungal cell component, and a fluorescent dye. The fraction of cells engulfing the particles was determined by flow cytometry as the dye-positive fraction. The results (Figure 2B) indicated that ^89^Zr-oxine labeling did not compromise phagocytic function. More than 71% of monocytes and over 85% of macrophages phagocytosed the particles, even at the higher ^89^Zr-labeling doses. As expected, B16 melanoma cells did not show phagocytosis. Overall, ^89^Zr-oxine labeling did not impair the functionality of monocyte–macrophage lineage cells in vitro.

### 2.4. ^89^Zr-Oxine PET/CT Visualized Trafficking of Monocytes and Macrophages in Mice

**Figure 3:**
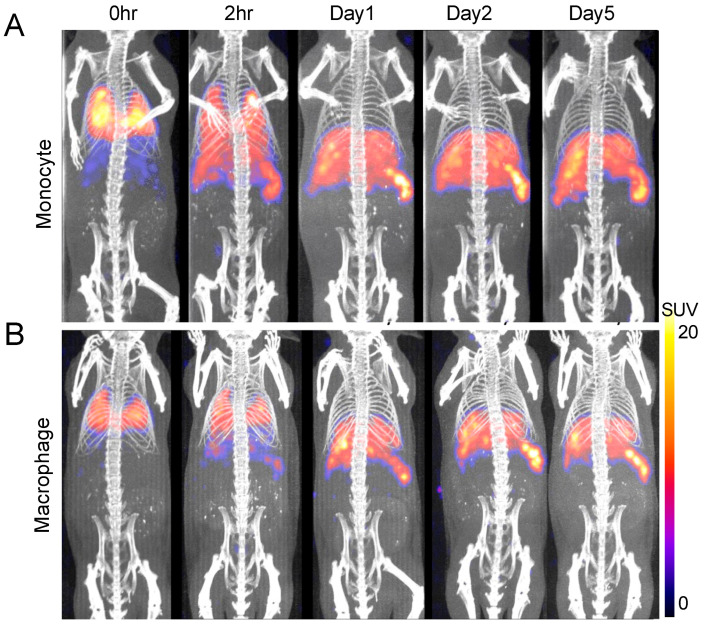
Serial PET/CT imaging demonstrates in vivo trafficking of ^89^Zr-oxine-labeled monocytes and macrophages in mice. (**A**,**B**) ^89^Zr-oxine-labeled monocytes ((**A**), 388.5 ± 21.4 kBq/10.5 million cells, *n* = 4) or ^89^Zr-oxine-labeled macrophages ((**B**), 247.9 ± 5.9 kBq/6.7 million cells, *n* = 3) were intravenously transferred into heathy mice. Representative serial maximum intensity projection PET images merged with CT are shown. Immediately post-injection, the infused cells localized to the lungs, followed by gradual redistribution to the liver and spleen by 2 h. A steady-state biodistribution pattern was observed by day 1 and persisted through day 7. Monocytes exhibited earlier clearance from the lungs compared to macrophages.

We then performed PET/computed tomography (CT) imaging of ^89^Zr-oxine-labeled monocytes (388.5 kBq/10.5 million cells) and macrophages (247.9 kBq/5.9 million cells) in heathy mice. Following intravenous infusion, the cells initially accumulated in the lungs, then progressively migrated to the liver and the spleen within 24 h, where they remained throughout the 5-day tracking period (Figure 3). Monocytes exhibited faster trafficking from the lungs to the liver and spleen compared to macrophages, as observed in the 2 h post-injection images.

### 2.5. Monocytes, but Not Macrophages, Are Recruited to Cutaneous Inflammation

**Figure 4:**
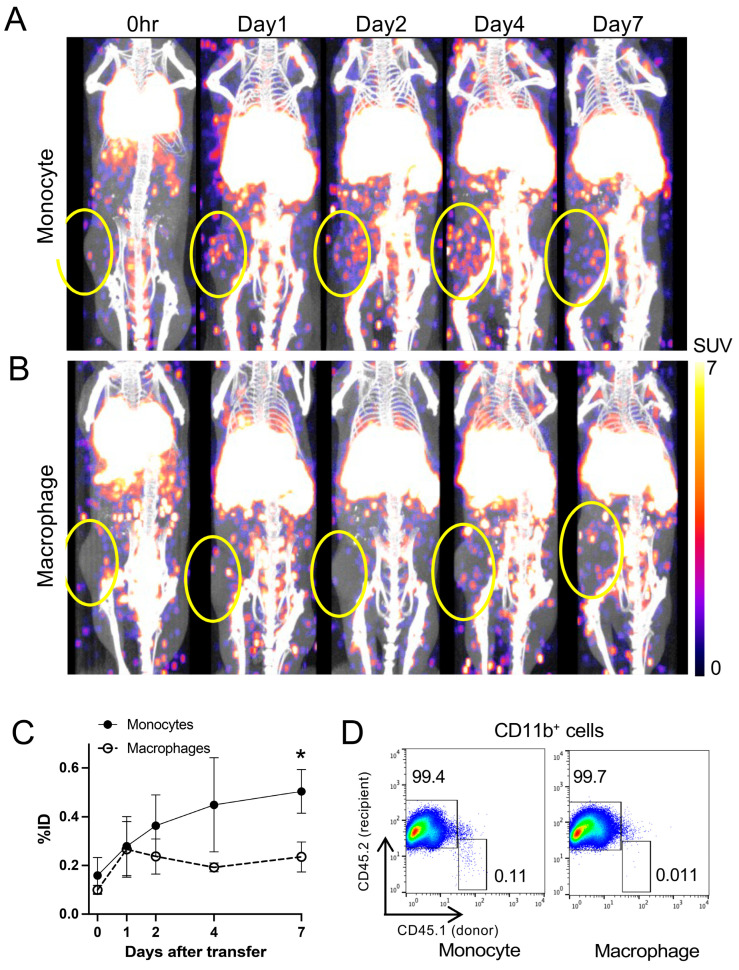
^89^Zr-labeled monocytes demonstrate greater trafficking to inflammatory granulomas than macrophages. (**A**,**B**) ^89^Zr-oxine-labeled monocytes ((**A**), 256.9 ± 69.5 kBq/8.5 million cells, *n* = 4) or macrophages ((**B**), 275.5 ± 58.0 kBq/8.5 million cells, *n* = 3) were intravenously transferred to mice bearing an inflammatory granuloma. Granulomas were generated by subcutaneous injection of polyacrylamide gel 1 day prior to the imaging. Representative serial maximum intensity projection PET images merged with CT show moderate accumulation of labeled monocytes at the granuloma site over time, whereas macrophages display minimal accumulation (circles indicate granuloma). (**C**) Quantitative analysis of the acquired PET images indicates the decay-corrected %ID of monocytes homed to the granuloma was significantly higher than that of macrophages on day 7 but also plateaued around day 7 (*: *p* < 0.05, repeated-measures two-way ANOVA). Data are presented as mean ± standard deviation. (**D**) Flow cytometry analysis of cells collected from the granulomas on day 5 confirmed higher frequencies of transferred monocytes (CD45.1^+^CD45.2^−^) within the total CD11b^+^ population compared to transferred macrophages (CD45.1^+^CD45.2^−^), supporting the PET results. Representative data are shown.

To assess trafficking to inflammatory lesions, a cutaneous granuloma was induced in mice and the migration of ^89^Zr-oxine-labeled monocytes and macrophages was evaluated. PET/CT imaging demonstrated a moderate accumulation of ^89^Zr-oxine-labeled monocytes (256.9 kBq/8.5 million cells) but not macrophages (275.5 kBq/8.5 million cells) in the granuloma (Figure 4A,B). We quantified the uptake on the acquired PET images and calculated the percentage of decay-corrected activity accumulated in the granuloma (percent injected dose, %ID) normalized to a 20 g mouse. The results indicated increasing monocyte accumulation in the inflammatory granuloma up to around day 4 and plateauing around day 7, whereas macrophage homing to the lesion was low and ceased as early as day 1 (Figure 4C). Consequently, monocyte accumulation was significantly higher than macrophage accumulation on day 7 (*p* < 0.05). Flow cytometry analysis of the cells collected from the granuloma on day 5 confirmed homing of transferred ^89^Zr-oxine-labeled monocytes to the inflammatory lesions, whereas the accumulation of macrophages was negligible (Figure 4D), consistent with the PET findings.

### 2.6. Monocytes Home to Cancers Better than Macrophages

**Figure 5:**
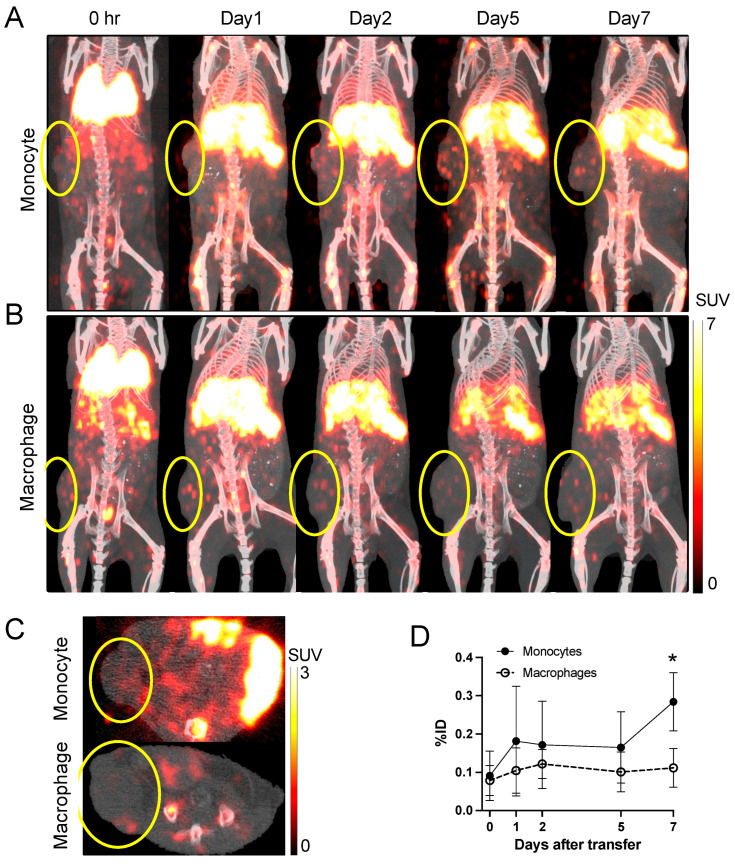
PET/CT imaging demonstrates greater trafficking of ^89^Zr-oxine-labeled monocytes to MC38 colon cancer than macrophages. (**A**,**B**) ^89^Zr-oxine-labeled monocytes ((**A**), 317.9 ± 77.6 kBq/9.9 ± 2.3 million cells, at 32.8 ± 8.4 kBq/million cells, *n* = 5) or macrophages ((**B**), 240.3 ± 116.1 kBq/8.7 ± 4.9 million cells, at 29.1 ± 6.6 kBq/million cells, *n* = 7) were intravenously transferred to mice bearing subcutaneous MC38 colon cancer. Representative maximum intensity projection PET images merged with CT are shown. Moderate accumulation of the monocytes was observed in the tumor, while macrophages showed minimal localization. Tumor sites are indicated by the circles. (**C**) Representative axial PET/CT images on day 2 demonstrating tumor-associated ^89^Zr-oxine-labeled cells. Tumor sites are indicated by the circles. (**D**) Quantitative analysis of PET images shows the significantly higher decay-corrected %ID in tumors of monocyte-injected mice compared to macrophage-injected mice on day 7 (*: *p* < 0.05, repeated-measures two-way ANOVA). Monocytes homed to the tumor significantly more than macrophages. Data are presented as mean ± standard deviation.

The successful visualization of monocyte trafficking to inflammatory lesions prompted us to investigate the homing of monocytes and macrophages to cancers, whose microenvironments are known to exhibit varying degrees of inflammation. Using a syngeneic subcutaneous MC38 murine colon cancer model, we intravenously transferred ^89^Zr-oxine-labeled monocytes (317.9 ± 77.6 kBq/9.9 ± 2.3 million cells) and macrophages (240.3 ± 116.1 kBq/8.7 ± 4.9 million cells), and tracked their migration by PET/CT imaging. Consistent with the observations in the granuloma model, ^89^Zr-oxine-labeled monocytes showed increasing tumor homing over time, while macrophage accumulation remained minimal (Figure 5A–C). Quantitative analysis of the acquired images indicated significantly greater monocyte infiltration into the tumor compared to macrophages on day 7 (*p* < 0.05) (Figure 5D). We observed similar results in another syngeneic tumor model of intramuscular B16 melanoma. Monocytes (injected at a dose of 388.6 kBq/8.5 million cells) again exhibited greater tumor recruitment than macrophages (injected at 342.9 kBq/8.5 million cells) (Figure S2). Although the %ID values quantified on the acquired images did not significantly differ between monocytes and macrophages, likely due to the limited number of animals evaluated (Figure S2C), flow cytometry analysis identified a higher fraction of transferred monocytes within the CD11b⁺ myeloid cell population compared to macrophages (Figure S2D).

**Figure 6:**
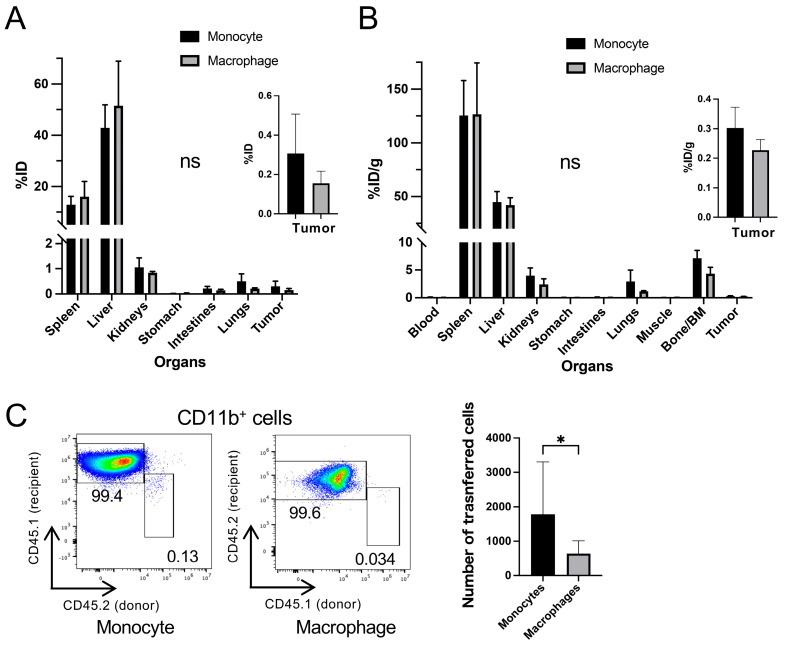
^89^Zr-oxine-labeled monocytes home more efficiently to MC38 colon cancers than macrophages, though the majority of the cells accumulate in the liver and spleen. (**A**,**B**) Biodistribution of ^89^Zr-oxine-labeled monocytes (303.8 ± 68.3 kBq/7.6 ± 1.4 million cells, at 40.1 ± 4.5 kBq/million cells, *n* = 6) and macrophages (179.5 ± 2.0 kBq/5.3 million cells, at 34.3 kBq/million cells, *n* = 6) 8 days after intravenous transfer to MC38-bearing mice was examined. Decay-corrected %ID (**A**) and %ID/g (**B**) values were normalized to a 20 g mouse (ns: not significant, two-way ANOVA). Tumor data are magnified and inset within the main graphs. (**C**) Labeled CD45.1^−^CD45.2^+^ monocytes were transferred to CD45.1^+^CD45.2^−^ recipient mice bearing MC38 tumors (266.4 ± 34.8 kBq/11.3 ± 1.4 million cells, at 24.3 ± 0.9 kBq/million cells, *n* = 8), and labeled CD45.1^+^CD45.2^−^ macrophages were transferred to CD45.1^−^CD45.2^+^ recipients (266.4 ± 34.8 kBq/11.3 ± 1.4 million cells, at 21.6 ± 2.5 kBq/million cells, *n* = 9) intravenously. Flow cytometry analysis performed 8 days post-transfer revealed a higher proportion of transferred monocytes within the CD11b^+^ population found in MC38 tumors than that of macrophages (left and middle, representative data). Absolute counts of transferred cells recovered from tumors confirmed significantly greater monocyte homing than that of macrophages (right, *: *p* < 0.05, unpaired Student’s *t*-test). All data in the graphs are shown as mean ± standard deviation.

We further examined the distribution of monocytes and macrophages in MC38-bearing mice. ^89^Zr-oxine-labeled cells were transferred intravenously (303.8 ± 68.3 kBq/7.6 ± 1.4 million monocytes or 179.5 ± 2.0 kBq/5.3 million macrophages), and 8 days later, organs and tissues were harvested for distribution analysis based on the ^89^Zr activity. Both monocytes and macrophages showed the highest %ID values in the liver, followed by the spleen (Figure 6A). When normalized by tissue weight, the highest %ID/g values were observed in the spleen, then in the liver (Figure 6B). The activity found in the bone/bone marrow likely reflects incorporation of free ^89^Zr released by cell death. Although monocytes showed higher %ID and %ID/g values in tumors than macrophages, consistent with PET image findings (Figure 5C), these differences were not statistically significant. This prompted us to determine the number of transferred cells homing to the MC38 tumors. Cell counting confirmed significantly higher monocyte infiltration into MC38 tumors than macrophages (*p* < 0.01, Figure 6C) 8 days post-transfer (monocytes at 266.4 ± 34.8 kBq/11.3 ± 1.4 million cells; macrophages at 209.8 ± 37.5 kBq/9.7 ± 0.6 million cells).

## 3. Discussion

In this study, we investigated the application of the ^89^Zr-oxine cell tracking method to monocytes and macrophages. The ^89^Zr-oxine complex has been successfully used to track a range of cell types by PET imaging, facilitating investigations into cellular responses to stimuli and the development of new cell-based therapies. Cell types previously investigated with this technique include various T cell subsets, chimeric antigen receptor (CAR)-expressing T cells, natural killer cells, DCs, B cells, eosinophils, and hematopoietic stem/progenitor cells [21,24,25,26,27,28,29,30,33]. The ^89^Zr-oxine labeling method offers a sensitive and quantitative means to detect transferred cells in vivo, with minimal radiotoxicity. Human dosimetry estimates derived from rhesus macaque PET imaging data demonstrated that the radio-exposures to normal organs and the whole body remain well below the established safety limit, owing to the extremely low radioactivity doses required for this cell tracking method [25]. However, despite these advantages, its application to monocyte–macrophage lineage cells has remained underexplored.

Recently, therapies based on monocytes and macrophages have garnered increasing attention for their potential to exert anti-tumor and anti-inflammatory effects [2,5,12,13,14], as well as for their drug-carrying capabilities. Some investigators attempted to exploit the phagocytic capability of macrophages by using them as live carriers of drug-containing nanoparticles to treat cancer [16,17]. A unique feature of monocytes and macrophages is their plasticity; monocytes can differentiate into DCs or macrophages, while macrophages can polarize into pro-inflammatory (M1) or anti-inflammatory (M2) phenotypes depending on environmental cues, making them attractive candidates as therapeutic cell products. Nevertheless, therapies based on monocytes and macrophages are not yet as well established as those employing T cells, DCs, or stem cells. Unlike T cell-based therapies, monocyte/macrophage-based approaches inherently lack antigen specificity. This limitation has been overcome by the recent development of chimeric antigen receptor-expressing macrophages (CAR-Ms), which impart antigen-specificity to macrophages [34]. CAR-Ms are capable of antigen-specific phagocytosis of cancer cells, and they undergo M1 polarization, producing pro-inflammatory cytokines, as a result of the viral vector used for transduction. Additionally, CAR-Ms can present antigens to T cells and convert tumor-resident M2 macrophages into the M1 phenotype [34,35]. These collective functions lead to significant tumor reduction in mouse models [34]. Importantly, CAR-M therapy mitigates a key concern of traditional monocyte/macrophage-based therapies, that the transferred cells might adopt an immune-suppressive M2 phenotype in the tumor microenvironment [34].

However, probably one of the most significant challenges is the low efficiency of monocyte/macrophage homing to target tissues following systemic administration. This limitation is often attributed to entrapment in the lungs and other reticuloendothelial organs [20]. Therefore, the ability to non-invasively and quantitatively monitor the real-time distribution of these cells is crucial for evaluating and improving the targeting capacity of these therapeutic cells.

We first evaluated the effects of ^89^Zr-oxine labeling on monocytes and macrophages in vitro and confirmed that labeling at doses sufficient for PET imaging did not impair viability, chemotaxis, or phagocytic function. These results and the labeling doses used (monocytes at 55.5 kBq/million cells and macrophages at 40.7 kBq/million cells) suggest that monocytes and macrophages are relatively radioresistant compared to stem cells and lymphocytes. For instance, T cells and bone marrow cells typically require labeling at less than 37 kBq/million cells to avoid radiotoxicity [21,24,29]. In the chemotaxis assays, ^89^Zr-oxine-labeled monocytes exhibited greater migration toward CCL2 compared to macrophages in a CCL2-dose-dependent manner. We demonstrated higher levels of CCR2 expression in monocytes compared to macrophages by flow cytometry, which supports monocyte’s higher chemotaxis toward CCL2. The CCL2/CCR2 axis is well known to mediate recruitment of CCR2-expressing cells to sites of inflammation and tumors, where CCL2 is upregulated [36]. Consistent with this, our PET/CT imaging showed greater accumulation of labeled monocytes than macrophages in both the inflammatory granuloma and two syngeneic cancer models: MC38 subcutaneous colon cancer and B16 intramuscular melanoma. However, the overall fraction of infused cells homing to these lesions was limited; less than 0.5% on day 7 or 8 after transfer in monocytes. The monocyte homing did not seem to increase substantially after these time points. Our results indicate that these cells will need further modification(s) to enhance their homing capability and improve therapeutic efficacy. Additionally, PET/CT imaging demonstrated that monocytes exited the lungs more rapidly than macrophages following intravenous administration. While this may partly be explained by the smaller size of monocytes compared to that of macrophages, the distinct migration kinetics are likely governed by a complex interplay of surface molecules, including adhesion molecules and chemokine receptors. Due to their relatively long retention in the lungs, macrophage-based cell therapy might be preferentially deployed in diseases of the lung.

While we successfully tracked monocytes and macrophages using ^89^Zr-oxine complex cell labeling and PET imaging, several limitations should be acknowledged. The macrophages used in this study were non-activated (M0) macrophages, and the monocytes we used consisted of mixed populations of Ly6C^high^ inflammatory monocytes and Ly6C^intermediate^ monocytes [37]. Recent studies have defined multiple macrophage subsets, such as M0, M1, and M2a-c, each characterized by distinct surface marker/phenotype profiles that reflect their activation and differentiation states [2,38]. Although various nomenclature systems are currently in use for classifying macrophage subsets, contributing to some confusion in the field [38], it remains an open and intriguing question whether these subsets exhibit unique trafficking behaviors in vivo. Indeed, previous reports indicate that M1 and M2 macrophages respond to different chemotactic cues [39], which could result in distinct in vivo trafficking patterns. Therefore, future studies should consider the impact of the macrophage polarization state on migration dynamics to better understand the diverse roles of these subsets in pathophysiological contexts. Although our results highlight the pivotal role of the CCL2/CCR2 axis in mediating monocyte migration, it is important to acknowledge the complexity of cell trafficking regulation, which involves multiple chemokine systems, adhesion molecules, and intracellular signaling pathways. Chemokine receptors such as CX3C motif chemokine receptor 1 (CX3CR1), selectins, and integrins, such as lymphocyte function-associated antigen 1 (LFA-1), may act synergistically with CCL2/CCR2 to facilitate monocyte recruitment, particularly in tissue- or lesion-specific contexts [40]. Further studies are required to elucidate how these pathways interact with CCR2-mediated signaling in coordinating monocyte migration in vivo. Additionally, vascularity of the lesions likely influences monocyte recruitment, as increased vascular perfusion and permeability can enhance immune cell infiltration. The relatively early monocyte homing to inflammatory granulomas and tumors observed in our models suggests the importance of perfusion in the lesions. This contrasts with clinical conditions such as abscesses, where the central regions are often poorly perfused and may not permit efficient monocyte trafficking. Therefore, it should be noted that our models represent an inflammatory environment with relatively preserved vascularity, rather than one with severely impaired perfusion.

^89^Zr-oxine PET/CT should be viewed in the context of other cell tracking methods such as bioluminescence imaging, fluorescence imaging, and magnetic resonance imaging (MRI) using iron nanoparticles [41]. Bioluminescence imaging requires the transfection of an enzyme or other catalytic molecule and the use of a foreign substrate, neither of which is easily translated into the clinic. Fluorescence imaging is limited by the shallow penetration depth of light in tissue, particularly in large animals and humans. Iron nanoparticle-loaded MRI, while capable of whole-body imaging, is insensitive for transferred cell localization and detection due to poor target to background ratios. Also, the iron-laden cells gradually release their payload, and endogenous phagocytes, such as macrophages, engulf and retain the released iron particles, potentially causing misleading false-positive signals [42,43,44]. Additionally, the doses of iron needed are of potential concern in the context of repeated imaging. In contrast, ^89^Zr-oxine PET imaging requires low radioactivity doses, offers high sensitivity, and enables whole-body imaging while providing a quantitative assessment of organ distribution [41].

In conclusion, we demonstrate that the ^89^Zr-oxine cell labeling with PET imaging enables reliable, non-invasive tracking of both monocytes and macrophages for at least one week, offering valuable insights into their biodistribution and trafficking dynamics. Importantly, ⁸⁹Zr-oxine labeling preserves cell viability and function, validating its utility for longitudinal cell tracking. We show the preferential recruitment of monocytes to both inflammatory and tumor lesions compared to macrophages, highlighting their greater mobility and potential superiority as therapeutic agents or delivery vehicles targeting inflammation and cancer. Our findings underscore the importance of cell lineage selection in monocyte–macrophage-based therapeutic strategies and provide a foundation for optimizing such approaches for future clinical applications.

## 4. Materials and Methods

### 4.1. Monocyte and Macrophage Differentiation, Cell Lines, and Cell Culture

All cells were cultured in RPMI 1640 medium supplemented with 2 mmol/L L-glutamine, 100 IU/mL penicillin/100 µg/mL streptomycin (all from Thermo Fisher Scientific, Waltham, MA, USA), 10% fetal calf serum (Gemini Bio Products, Sacramento, CA, USA), and 50 µmol/L 2-mercaptoethanol (Sigma-Aldrich, St. Louis, MO, USA) at 37 °C in 5% CO_2_. Viable cells were counted using either a Countess Automated Cell Counter and 0.4% trypan blue dye (Thermo Fisher Scientific) or a LUNA-FL Dual Fluorescence Cell Counter and Acridine Orange/Propidium Iodide Stain (Logos Biosystems, Anyang-si, South Korea), following the manufacturers’ instructions.

Monocytes and macrophages were differentiated from bone marrow cells flushed out of the femurs and tibias of mice, using recombinant murine macrophage-colony stimulating factor (M-CSF, PeproTech, Cranbury, NJ, USA) at 20 ng/mL for 5–6 days and at 10 ng/mL for 7–10 days, respectively. Ultra-low attachment flasks (Corning; Corning, NY, USA, or Nalge Nunc International, Rochester, New York, NY, USA) were used for all the monocyte cultures, and non-adherent cells were collected for downstream applications (as attached cells differentiate into macrophages). For macrophages, which adhered firmly to the flask surface, cells were detached using the non-enzymatic cell dissociation solution Cellstripper (Corning) and collected gently with a cell scraper (Thermo Fisher Scientific). B16 murine melanoma cells were purchased from the American Type Culture Collection (Manassas, VA, USA), and MC38 murine colon cancer cells were obtained from Kerafast (Boston, MA, USA).

### 4.2. Mouse Granuloma and Tumor Models

Animal experiments were performed in accordance with a protocol approved by the National Cancer Institute Animal Care and Use Committee. C57BL/6 wild-type (CD45.1^−^CD45.2^+^) and congenic (CD45.1^+^CD45.2^−^) mice were purchased from Jackson Laboratory (Bar Harbor, ME, USA). Male and female mice aged 10 to 24 weeks were used in the experiments. Where applicable, mice were randomly assigned to groups, and gender-matched mice were used for cell transfer studies.

Granulomatous inflammation was induced by subcutaneous injection of a mixture of polyacrylamide gel (PAG) pellets (Bio-Gel P-100 Gel, Bio-Rad Laboratories, Hercules, CA, USA) and lipopolysaccharide (LPS, Sigma-Aldrich) (3.5% PAG in 1 mL PBS and 10 µg LPS) [45,46] into the right flank 24 h prior to monocyte or macrophage transfer. For tumor models, mice were injected with 4 × 10^6^ B16 cells intramuscularly or 1.2 × 10^6^ MC38 cells subcutaneously into the right hind leg. Tumors were allowed to develop for 7 days (B16) or 10 days (MC38), respectively, before cell transfer.

### 4.3. Flow Cytometry Analysis

Monocytes and macrophages were pre-incubated with anti-mouse CD16/32 antibody (clone 93, eBioscience/Thermo Fisher Scientific, San Diego, CA, USA) for 15 min at room temperature to block Fc receptor binding. Surface marker staining was then performed for 30 min at 4 °C. Fluorochrome-conjugated anti-mouse antibodies against CD11b (clone M1/70), F4/80 (clone BM8), CD80 (clone 16-10A1), Ly-6C (clone HK1.4), Ly6G (clone 1A8-Ly6g), CD11c (clone N418), CD80 (clone 16-10A1), CD45.1 (clone A20), and CD45.2 (clone 104) were purchased from eBioscience/Thermo Fisher Scientific. Antibodies against CD62L (clone MEL-14, BioLegend, San Diego, CA, USA) and CCR2 (clone 475301, R&D Systems, Minneapolis, MN, USA) were also purchased. For MC38 tumor-infiltrating cell analysis, a Fixable Viability Dye eFluor 455UV (eBioscience/Thermo Fisher Scientific) was applied prior to Fc receptor blocking and surface staining. The stained cell samples were applied to a FACSCalibur flow cytometer (Becton Dickinson, Franklin Lakes, NJ, USA) or a CytoFLEX LX flow cytometer (Beckman Coulter, Brea, CA, USA). The obtained data were analyzed using FlowJo software version 10.0.8, or version 10.10.0 (Becton Dickinson), with appropriate gating for live, single cells. The flow cytometry methods related to phagocytosis assays can be seen below.

### 4.4. ^89^Zr-Oxine Synthesis and Cell Labeling

^89^Zr-chloride (^89^ZrCl_4_) was prepared from ^89^Zr-oxalate generated at the cyclotron facility (Clinical Center, National Institutes of Health, Bethesda, MD, USA) and was used for the synthesis of ^89^Zr-oxine, as previously described [21,47]. To summarize, 2 µL of 20% Tween 80 solution, 102 µL of 20 mmol/L oxine in 0.04 N HCl, and 60 µL of ^89^ZrCl_4_ in 1 N HCL were added sequentially, and then, the solution was neutralized by adding increments of 500 mmol/L NaHCO_3_ until the pH reached 7.0–7.5. Monocytes and macrophages were incubated with ^89^Zr-oxine at a standard dose of 74 kBq per million cells (unless otherwise specified) in PBS for 15 min, followed by two washes in complete medium. The labeled cells were then transferred to a fresh tube and washed one more time. The final labeled activity was measured using a dose calibrator (CRC-25W, Capintec, Ramsey, NJ, USA).

### 4.5. Cell Viability and ^89^Zr Retention After Labeling

Monocytes and macrophages were labeled with ^89^Zr-oxine at two different doses, a standard imaging dose (approximately 37 kBq/million cells) and a high dose (355.9–732.6 kBq/million cells), which was more than 10-fold higher than the imaging dose. Both labeled and unlabeled cells were cultured with M-CSF (PeproTech) in 6-well plates, and the number of viable cells was determined using a Countess Automated Cell Counter and 0.4% trypan blue dye (both from Thermo Fisher Scientific) at designated time points up to day 7. To assess ^89^Zr retention, cell suspensions were centrifuged (1900× *g* for 2 min) and the radioactivity of the cell pellets was measured by a WIZARD2 automatic gamma counter (Perkin Elmer, Waltham, MA, USA).

### 4.6. Chemotaxis Assay

The migration of non-labeled and ^89^Zr-oxine-labeled monocytes (55.5 kBq/million cells) and macrophages (38.1 kBq/million cells) toward CCL2 was examined using transwell inserts with a 5 μm pore size (Millicell, Millipore, Billerica, MA, USA) placed in 12-well plates (Corning). A total of half a million cells were seeded into the upper chamber, while the lower chambers were filled with culture medium containing 10 or 100 ng/mL recombinant murine CCL2 (PeproTech). After 24 h of culture, cells that had migrated into the lower chambers were harvested and counted.

### 4.7. Phagocytosis Assay

Unlabeled and ^89^Zr-oxine-labeled monocytes and macrophages (at indicated doses) were incubated for 2 h with zymosan particles conjugated with a pH-sensitive fluorescent dye (pHrodo Green Zymosan Bioparticles Conjugate for Phagocytosis, Thermo Fisher Scientific). The dye remains non-fluorescent at neutral pH but fluoresces in the acidic environment of phagosomes, allowing quantification of active phagocytosis. B16 melanoma cells were included as a non-phagocytic cell control. After 24 h of incubation, the fraction of cells that engulfed the zymosan particles was assessed by a FACSCalibur flow cytometer (Becton Dickinson) and analyzed with FlowJo software version 10.0.8 (Becton Dickinson).

### 4.8. Tracking Monocytes and Macrophages by PET/CT Imaging

^89^Zr-oxine-labeled monocytes or macrophages were intravenously transferred into mice at the indicated labeling doses and cell numbers. To reduce bone uptake of free ^89^Zr potentially released from dying labeled cells, an iron chelator deferoxamine (Hospira, Inc., Lake Forest, IL, USA) was injected intramuscularly at a dose of 660 µg, 15 min prior to cell transfer, and again at 1, 2, 3, and 4 h, as well as 1-day post-transfer, to enhance renal excretion of ^89^Zr. Imaging was performed using a microPET/CT imager (BioPET, Bioscan, Washington, DC, USA) for up to 7 days. Images were acquired immediately after cell transfer (5 min per bed position, 2 bed positions), with extended acquisition times at subsequent time points to account for radioactive decay. PET images, including maximum intensity projections, were merged with CT images using Vivo Quant software (version 2.0, inviCRO LLC, Needham Heights, MA, USA) or MIM software (version 7.3.5, MIM Software, Cleveland, OH, USA). Regions of interest were manually drawn on the PET/CT sections outlining the tumors or inflammatory lesions, and the radioactivity within these volumes of interest was quantified.

### 4.9. Determination of Recruitment of Transferred Monocytes and Macrophages to Granulomas and Tumors

To determine the cells infiltrated into the cutaneous granulomas or tumors, mice were sacrificed by CO_2_ inhalation and the granulomas or tumors were harvested (2 days after cell transfer for the granuloma and B16 melanoma models, and 8 days after cell transfer for the MC38 model). Single cell suspensions were made from these tissues by passing the cut tissue through a 70 µm nylon strainer in PBS with or without digestion of the tissue by 50 µg/mL Liberase TM (Roche, Basel, Switzerland) for 30 min at 37 °C. The cells were counted, washed by PBS with 0.1% fetal calf serum, and stained for surface antigen expression for flow cytometry analyses. To distinguish injected cells from endogenous monocyte–macrophage linage cells, we used a CD45.1/CD45.2 congenic mouse system. The number of transferred cells in the tumors was determined by multiplying their percentage among all recovered cells by the total cell count following tumor tissue digestion.

For biodistribution studies in the MC38 model, ^89^Zr-oxine-labeled monocytes (303.8 ± 68.3 kBq/7.6 ± 1.4 million cells, at 40.1 ± 4.5 kBq/million cells) or macrophages (179.5 ± 2.0 kBq/5.3 million cells, at 34.3 kBq/million cells) were intravenously transferred. Eight days later, mice were euthanized and weighed, blood was collected, and indicated organs and tissues were harvested. Each organ/tissue was weighed, and its ^89^Zr activity was measured using the gamma counter. The percent injected dose (%ID), normalized to a 20 g mouse, was calculated using the following formula: %ID = [decay-corrected radioactivity of the tissue (cpm)]/[injected activity (cpm)] × [body weight (g)]/20 (g). The percent injected dose per gram of tissue (%ID/g), normalized to a 20 g mouse, was calculated as follows: %ID/g = [decay-corrected radioactivity of the tissue (cpm)]/[injected activity (cpm)]/[tissue weight (g)] × 100 × [body weight (g)]/20 (g), where cpm refers to the count per minute.

## 5. Statistical Analysis

All statistical analyses were performed using Prism software (version 10.5.0, Graph-Pad Software, La Jolla, CA, USA). For comparisons between two groups involving a single variable, two-tailed unpaired Student’s *t*-tests were used. For comparisons involving multiple groups and two variables, a two-way analysis of variance (ANOVA) was applied. When evaluating two variables across multiple time points, a repeated-measures two-way ANOVA was used. A *p*-value less than 0.05 was considered statistically significant.

## Data Availability

The original contributions presented in the study are included in the article and Supplementary Materials, further inquiries can be directed to the corresponding author.

## Supplementary Materials

The following supporting information can be downloaded at https://www.mdpi.com/article/10.3390/ph18060897/s1: Figure S1: Bone marrow-derived monocytes and macrophages exhibit distinct surface marker profiles. Figure S2: ^89^Zr-oxine-labeled monocytes home to B16 melanoma more than macrophages.
