# Supplementary material for: Zirconium-89-Oxine Cell Tracking by PET Reveals Preferential Monocyte Recruitment to Cancer and Inflammation over Macrophages
Source: Pharmaceuticals (Basel). 2025 Jun 15;18(6):897. doi: 10.3390/ph18060897 (PMC12196144; doi:10.3390/ph18060897)
Supplement: Supplementary file 1 [file pharmaceuticals-18-00897-s001.zip › pharmaceuticals-3664982-supplementary.pdf]

Supplementary Materials for

Zirconium-89-Oxine Cell Tracking by PET Reveals Preferential Monocyte Recruitment to Cancer and Inflammation over Macrophages

Sho Koyasu [1,2,†], Hannah A. Minor [1,3,†], Kingsley O. Asiedu [1,‡], Peter L. Choyke [1] and Noriko Sato [1,*]

[1] Molecular Imaging Branch, National Cancer Institute, National Institutes of Health, Bethesda, MD 20892, USA

[2] Department of Diagnostic Imaging and Nuclear Medicine, Graduate School of Medicine, Kyoto University, Kyoto 606-8507, Japan

[3] School of Arts and Sciences, University of Maryland Global Campus, Adelphi, MD 20783, USA

[*] Correspondence: saton@mail.nih.gov; Tel.: +1-240-858-3079; Fax: +1-240-541-4526

[†] These authors contributed equally to this work.

[‡] Current affiliation: Department of Radiology, Duke University Medical Center, Durham, NC 27710, USA.

Supplementary Figures

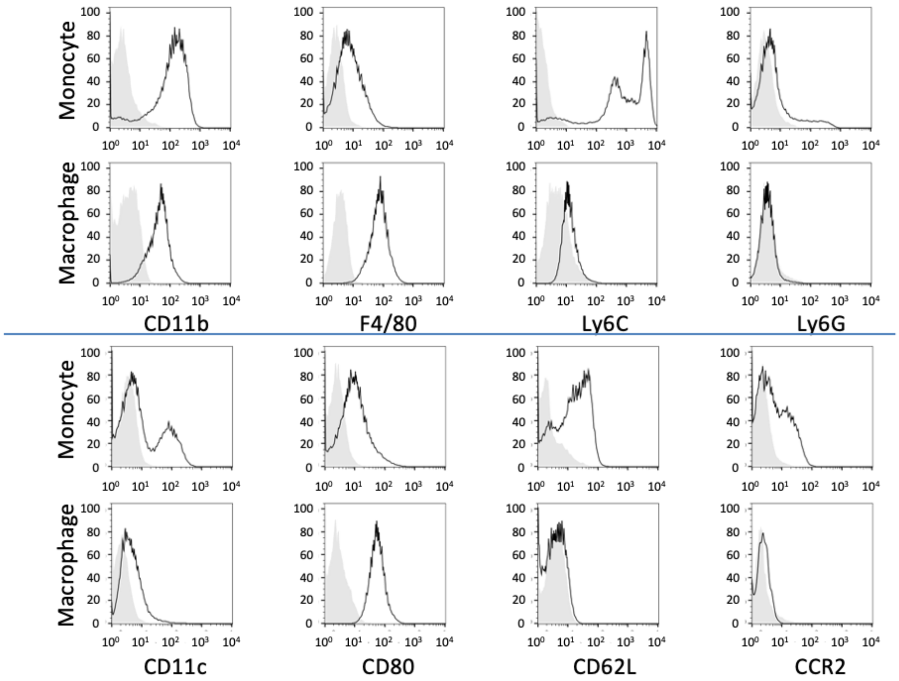

Figure S1. Bone marrow–derived monocytes and macrophages exhibit distinct surface marker profiles. Monocytes differentiated with M-CSF for 5 days and macrophages differentiated for 7 days were analyzed by flow cytometry for the indicated surface markers. Solid lines represent specific antibody staining; shaded histograms indicate isotype controls. Representative data from more than two independent experiments are shown.

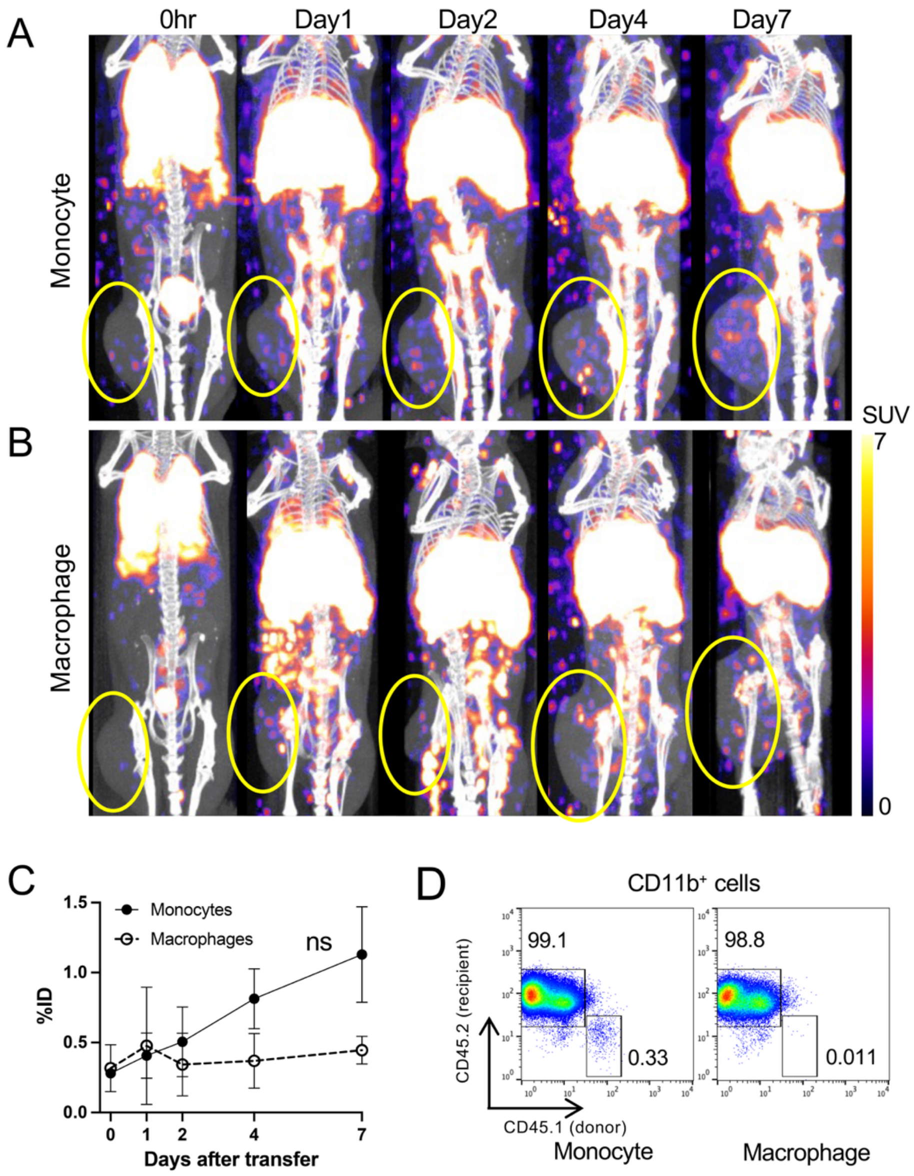

Figure S2. ^{89}Zr-oxine labeled monocytes home to B16 melanoma greater than macrophages. (**A, B**) ^{89}Zr-oxine labeled monocytes (**A,** 388.6 ± 33.6 kBq/8.5 million cells, n = 4) or macrophages (**B**, 342.9 ± 59.2 kBq/8.5 million cells, n = 3) were intravenously transferred to mice bearing intramuscular B16 melanoma. Representative maximum intensity projection PET images overlaid with CT are shown. Monocytes exhibited moderate and increasing tumor accumulation over time, whereas macrophages showed minimal localization until day 7. B16 tumor sites are indicated by the circles. (**C**) Quantitative analysis of PET images revealed higher decay-corrected %ID in the tumors of monocyte-injected mice compared to macrophage-injected mice, although the

difference did not reach statistical significance (ns: not significant, repeated-measures two-way ANOVA). Data are presented as mean ± standard deviation. (**D**) Flow cytometry analysis of cells collected from the tumors on day 5 indicated a higher proportion of transferred monocytes ($CD45.1^{+}CD45.2^{-}$) within the total $CD11b^{+}$ population compared to transferred macrophages ($CD45.1^{+}CD45.2^{-}$), supporting the imaging findings. Representative data are shown.
